# Reduced vancomycin susceptibility in porcine ST9 MRSA isolates

**DOI:** 10.3389/fmicb.2013.00316

**Published:** 2013-10-25

**Authors:** Gabriella M. L. Kwok, Margaret M. O'Donoghue, Vijaya C. Doddangoudar, Jeff Ho, Maureen V. Boost

**Affiliations:** ^1^Department of Health Technology and Informatics, The Hong Kong Polytechnic UniversityKowloon, Hong Kong; ^2^School of Nursing, The Hong Kong Polytechnic UniversityKowloon, Hong Kong

**Keywords:** livestock-associated MRSA, vancomycin, vraS, graR, spiral gradient endpoint technique, VRSA, hVISA

## Abstract

Porcine strains of livestock-associated methicillin resistant *Staphylococcus aureus* (LA-MRSA) have been recognized in many countries and have been shown to be able to cause human infection. Resistance to non-beta lactam antibiotics has been reported but non-susceptibility to vancomycin, which is known to occur in human MRSA, has so far not been observed in LA-MRSA. Such resistance is typically fairly low level involving changes in the cell wall thickness. The development of resistance is usually preceded by presence of a sub-population having an increased MIC, which is selected for by exposure to vancomycin. This study investigated vancomycin susceptibility of one hundred porcine MRSA isolates using three MIC methods including spiral gradient endpoint (SGE) technique which allows visualization of more resistant sub-populations. SGE revealed 16 strains with an MIC above 2.0 mg/L, of which 14 were determined to have MIC 4 mg/L by agar dilution (AD). SGE revealed a further two isolates with MIC < 2 mg/L had a sub-population >2 mg/L. In addition, trailing endpoints not reaching resistance were present in 26 isolates with MIC < 2 mg/L. Sequencing of the genes of the VraSR/GraSR two component systems of ten of the resistant strains for comparison with susceptible strains revealed changes, including the presence of stop codons, in *vra*S and *gra*R, but these were not consistent in all isolates. Other genetic changes may contribute to vancomycin non-susceptibility and require investigation. As failure to respond to treatment has been reported in clinical isolates with MIC > 1.5 mg/L, the presence of vancomycin non-susceptibility in porcine isolates is of concern and further monitoring of LA-MRSA is essential.

## Introduction

Methicillin resistant *Staphylococcus aureus* (MRSA) has long been an important hospital-associated pathogen, but its emergence in the community and, more recently, in livestock has led to increasing concern. Livestock-associated MRSA (LA-MRSA), first recognized in the Netherlands (Voss et al., 2005), has now been reported from many locations, including Asia (Denis et al., 2009; Guardabassi et al., 2009; Smith et al., 2009; Larsen et al., 2012). However, Asian isolates differ from those of Europe, belonging almost exclusively to ST9 (Cui et al., 2009; Guardabassi et al., 2009; Neela et al., 2009), rather than ST398 which is the most prevalent lineage in Europe and North America. In addition, Asian isolates are more likely to be multi-drug resistant, displaying resistance to up to eight agents (Ho et al., 2012). To date it appears that LA-MRSA strains are less virulent than human isolates and, although these strains do colonize and occasionally cause infections in man, their human-to human transmission risk is much lower than that of typical MRSA (Bootsma et al., 2011). However, their ability to cause infection does increase concern, as their antibiotic resistance profile is extensive leading to limited choices of therapy. To date there have not been any reports of resistance to vancomycin in LA-MRSA, though non-susceptibility to this drug has frequently been reported in typical human MRSA isolates (Howden et al., 2010).

Most vancomycin resistance in *S. aureus* involves changes that result in thickening of the bacterial cell wall, sometimes termed intermediate resistance, rather than by acquisition of the *van*A gene observed in high level resistance (Howden et al., 2010). The thickening of the cell wall has been attributed to mutations of regulatory genes including those of the two component systems *vra*SR/*gra*SR, and *wal*K as well as *rpo*B, with a large number of the studies reporting changes in *vra*S and *gra*R (Neoh et al., 2008; Cui et al., 2009; Doddangoudar et al., 2011, 2012; Galbusera et al., 2011). Vancomycin intermediately-resistant *S. aureus* (VISA), has been redefined as vancomycin resistant *S. aureus* (VRSA) since the revision of the European Committee for Antimicrobial Susceptibility Testing guidelines (EUCAST, 2013). The change was justified by the recognition of treatment failures in infections attributable to isolates with MIC of 2 mg/L (Lodise et al., 2008). Strains with more resistant sub-populations, first recognized in Japan (Hiramatsu et al., 1997), are referred to as heterogeneous vancomycin intermediately resistant *S. aureus* (hVISA) but are not clearly defined in EUCAST guidelines. Such strains may rapidly progress to VRSA in the presence of vancomycin (Howden et al., 2010).

Testing for vancomycin non-susceptibility requires accurate determination of the MIC. Many of the standard methods have been criticized either for their lack of accuracy or reproducibility or because the large increments between dilutions makes smaller changes more difficult to detect. The use of the spiral gradient endpoint (SGE) technique can overcome these difficulties and this method has been shown to produce reliable results with excellent correlation with the CLSI recommended dilution method (Doddangoudar et al., 2010).

This study determined the prevalence of vancomycin non-susceptibility in LA-MRSA isolates from slaughtered pigs and compared sequences of *vra*S and *gra*R of resistant isolates with those of both sensitive strains, a known vancomycin intermediately-resistant strain (Mu50), and a control vancomycin sensitive MRSA.

## Materials and methods

One hundred MRSA (ST9) previously isolated from slaughtered pigs originating in China (Ho et al., 2012) were investigated for reduced vancomycin susceptibility. Although, these strains had been previously tested for vancomycin resistance by broth microdilution (BMD), with none giving an MIC > 2 mg/L (Ho et al., 2012), later testing of a few isolates using agar dilution (AD) and SGE for a separate study suggested that some could have MICs above 2 mg/L or harbor a resistant sub-population. The strains, which had been stored at −80°C, were subcultured on brain heart infusion (BHI) agar and the MIC for vancomycin determined using the SGE technique (Doddangoudar et al., 2010) and AD (Clinical Laboratory Standards Institute, 2012).

SGE was performed by dispensing 50 μL of a 1072 mg/L vancomycin stock solution on a BHI agar plate using a spiral plater (Autoplate 4000, Spiral-Biotech, Advanced Instruments, Norwood, MA, USA) to produce a concentration gradient of 0.5–8 mg/L on each plate. The plates were allowed to rest for 1.5 h to allow the gradient to develop. Each porcine isolate was inoculated into 2 mL BHI broth and incubated at 37°C to achieve a turbidity equivalent to a 0.5 McFarland standard. These inocula were streaked across the prepared vancomycin gradients, each specimen being performed in duplicate as is standard practice for this method (Doddangoudar et al., 2010). The plates were incubated for 24 h and the endpoint of growth was determined using the SGE template available from Spiral Biotech. Control strains, N315 (vancomycin sensitive MRSA: VSSA), and NRS1 (Mu50: VISA) were included in every test batch. Both the confluent endpoint (EC) of growth and the presence and extent of any trailing endpoint (TEC) were noted. The EC concentration, which was equivalent to the vancomycin MIC, was determined using the SGE software (Spiral Biotech). A TEC, with higher inhibitory concentration, may indicate the presence of a more resistant sub-population of the strain as is reported in hVISA strains. MICs were interpreted for both AD and SGE using EUCAST guidelines (EUCAST, 2013). To confirm accuracy of SGE, 20 isolates were repeated in duplicate 1 week after completion of the initial MIC testing.

Glycopeptide resistance detection (GRD) Etest (Yusof et al., 2008) and macro Etest (MET) (Walsh et al., 2001) were used for the isolates with TECs as further tests to detect hVISA. AD and SGE were incubated for 24 h, but incubation was extended to 48 h for MET and GRD.

Following MIC determination, DNA was extracted from the 10 isolates with highest MICs. N315 and a vancomycin sensitive porcine isolate (MIC 1.0 mg/L) were included as controls. Both *vra*S and *gra*R were amplified as previously described (Doddangoudar et al., 2011) and visualized by agarose gel electrophoresis and ethidium bromide staining. The amplicons were cleansed by treatment with 0.05 μL of 20 U/μL exonuclease (Thermo-Fisher, Pittsburgh, PA, USA) and 1 μL of 1 IU/μL shrimp alkaline phosphatase (Thermo-Fisher) at 37°C for 30 min followed by 15 min at 80°C, before sequencing. Sequences from porcine isolates were compared with those of N315 and Mu50 using multiple sequence alignment software (Corpet, 1988), base changes being categorized as silent or non-synonymous mutations. Sequences of the vancomycin susceptible and non-susceptible porcine isolates were also compared to determine if there were any common changes between susceptible and non-susceptible isolates and amongst non-susceptible isolates.

## Results

**Figure 1:**
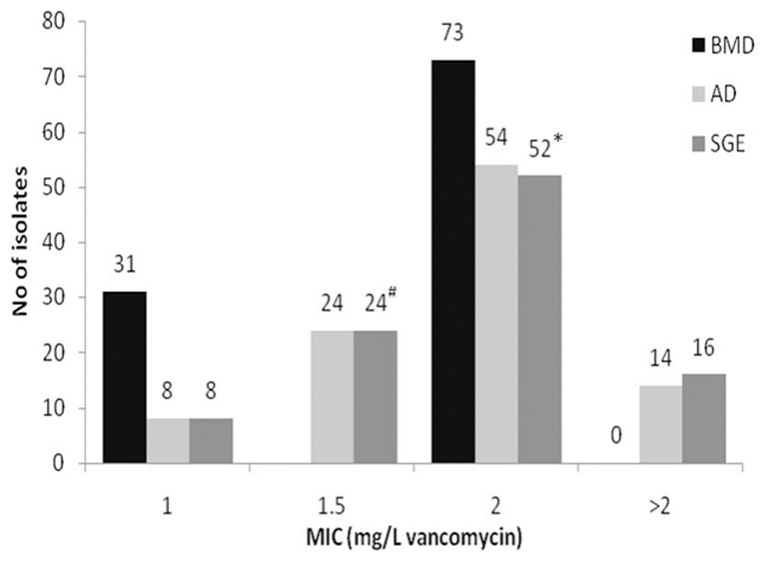
**Comparison of vancomycin MICs of porcine MRSA by broth microdilution (BMD) [from Ho et al. (2012)], agar dilution (AD), and spiral gradient endpoint technique (SGE)**. ^#^Two isolates displayed a sub-population above 2.0 mg/L (hVISA). ^*^Five isolates displayed sub-populations by Macro Etest; of these, three isolates displayed sub-populations by Glycopeptide Resistance Detection. The latter three strains displayed a TEC MIC for SGE of =2.5 mg/L, the remaining two had TEC < 2.5 mg/L.

Low level non-susceptibility was observed in 16 isolates which displayed MIC values ranging from 2.1 to 2.3 mg/L vancomycin by SGE. Of these, 14 isolates displayed growth on AD plates at 2 mg/L, thus, were reported as MIC 4 mg/L. The remaining two strains were inhibited, but grew well at 1 mg/L, thus, having an MIC of 2 mg/L by the standard method. Of the 16 strains having an elevated MIC, five displayed a TEC with a higher value indicating the presence of a more resistant sub-population. SGE revealed a further two isolates with an EC below two (1.8 mg/L) displayed TEC above 2 mg/L suggesting that these strains had a more resistant sub-population (Table 1). Testing of the five strains with MIC > 2 mg/L and displaying a TEC using GRD and MET did not result in any of these having an MIC ≥ 8.0 mg/L, but microcolonies were present in the clear area showing presence of more resistant sub-populations in all five by MET and three using GRD. Comparisons of MICs by BMD (Ho et al., 2012), AD, and SGE are shown in Figure 1.

**Table 1:** **Minimum inhibitory concentrations (MIC) to vancomycin of porcine MRSA isolates by SGE**.

**Classification**	**Number of isolates**	**Average MIC − EC**	**Range EC**	**Average MIC − TEC**	**Range TEC**	**MIC_50_**	**MIC_90_**
VSSA^a^	82	1.7	1.2–1.9	1.8^d^	1.5–1.9		
VSSA^c^	2	1.8	1.8	2.2	2.2		
VRSA^a^,^b^	16	2.1	2.1–2.3	2.5^e^,^f^	2.2–3.0		
Overall	100	1.8	1.2–2.3	1.9	1.5–3.0	1.8	2.1

EC—confluent endpoint concentration which represents the MIC of the majority of the population.

TEC—trailing endpoint concentration which represents the MIC of the sub-population.

a EUCAST definition (2009) sensitive (VSSA): ≤ 2 mg/L; resistant (VRSA): = MIC > 2.0 mg/L.

b 14 of these strains gave MIC 4 mg./L by AD.

c EC MIC ≤ 2 mg/L, TEC > 2 mg/L.

d Present in 26 isolates (not >2 mg/L).

e Present in 5 isolates, three of which were 2.5 mg/L. (all showed micro-colonies in MET).

f Three of these showed microcolonies in GRD.

In addition to the presence of resistant strains, the overall average MIC was relatively high (1.8 mg/L) and strains classed as VSSA had an average MIC of 1.7 mg/L (Table 1). TECs with MICs below 2 mg/L were present in 26/82 (32%) of the VSSA strains. The MIC values obtained for the control strains NRS1 and N315, which were performed for every batch tested, were in the expected range (8.5 and 1.1 mg/L) and repeated estimations of 20 of the samples showed <10% differences in MIC values, indicating SGE provided reliable and accurate results.

Visualization of the amplicons of the 10 samples with highest MIC values and the control strains revealed clear bands situated between 1000 and 1650 bp for *vra*S and between 650 and 850 bp for *gra*R on the DNA ladder which were similar to the target gene sizes of 1041 and 672 bp reported for these genes. Comparison of sequences revealed non-synonymous changes in *vra*S in three isolates. In M1 (VSSA) a deletion at base 741 resulted in a frameshift and generation of a novel stop codon at amino acid 278. In two of the resistant strains, M7 and M10, V275STOP, and N279I were observed, respectively (Table 2). Changes in comparison to the controls were also noted in *gra*R in the VSSA isolate, M1. There were four successive mutations at base numbers 672−5, leading to a E224D missense mutation, followed by three consecutive mutations leading to insertion of a codon coding for isoleucine, at the position where the stop codon should be thereby elongating the amino acid chain by one amino acid before the stop codon. Several mutations were observed in the non-susceptible strains, with V6L and E15K both present in four strains, and E120D in three. Stop codons were present in both M3 and M9 and other unique mutations were observed in M7 and M11 (Table 2).

**Table 2:** **Presence of mutations observed in one vancomycin susceptible (M1) and 10 vancomycin non-susceptible (M2–11) porcine isolates in comparison with N315 (control MRSA)**.

**Mutation**	**Sample number**										
**Sample**	M1	M2	M3	M4	M5	M6	M7	M8	M9	M10	M11
**Vancomycin MIC**	1.4	2.2	2.2	2.2	2.1	2.3	2.1	2.2	2.1	2.1	2.1
**By SGE (TEC)**	(-)	(3.0)	(-)	(-)	(2.5)	(-)	(2.5)	(-)	(2.2)	(-)	(-)
***vra*S**											
F278STOP	+	−	−	−	−	−	−	−	−	−
V275STOP	−	−	−	−	−	−	+	−	−	−	−
N279I	−	−	−	−	−	−	−	−	−	+	−
***graR***											
I206STOP	−	−	+	−	−	−	−	−	−	−	−
E224D	+	−	−	−	−	−	−	−	E224STOP	−	−
STOP225I	+	−	−	−	−	−	−	−	−	−	−
V6L	−	−	+	+	−	−	−	+	+	−	−
E19K	−	−	+	−	−	−	−	+	+	−	+
E120D	−	−	+	−	−	−	−	−	+	+	−
V216STOP	−	−	−	−	−	−	+	−	−	−	−
E15K	−	−	−	−	−	−	−	−	−	−	+
R117R	−	−	−	−	−	−	−	−	−	−	+
D133N	−	−	−	−	−	−	−	−	−	−	+
A185P	−	−	−	−	−	−	−	−	−	−	+
L203STOP	−	−	−	−	−	−	−	−	−	−	+

## Discussion

Whilst vancomycin non-susceptibility has been reported in several countries and there have been a number of reports of treatment failure for human clinical isolates, this study appears to be the first to describe reduced susceptibility in MRSA of porcine origin. There have been several reports describing human colonization and infection with porcine strains (Voss et al., 2005; van der Mee-Marquet et al., 2011) and elevated vancomycin MICs as observed in our isolates would render treatment with vancomycin more prone to fail (Lodise et al., 2008; Jacob and Diazgranados, 2013). Overall, 16% of isolates appeared to have reduced susceptibility with MICs above 2.0 mg/L using SGE, and 14% by AD. In addition, the overall average MIC was close to the breakpoint by all three MIC methods used. These strains should not have been exposed to glycopeptides, since use of avoparcin has been banned in China, as elsewhere, for more than a decade, due to concerns of its contribution to development of bacterial resistance (Witte, 1998). Reports of clinical infections caused by vancomycin-resistant enterococci in both humans and animals dropped dramatically in Europe and Taiwan after the ban (Sørum et al.,^2006^; Lauderdale et al., 2007). It is possible that glycopeptides other than avoparcin are in use thereby circumventing the ban on a specific agent. Alternatively, the feeding of mycelium, which is practiced in China, may provide antibiotic agents (Witte, 1998). However, it has also been suggested that use of antibiotics other than glycopeptides may lead to increases in resistance to vancomycin (Katayama et al., 2009; Tattevin et al., 2009). The high rate of non-susceptibility suggests that there has been some antibiotic exposure during production and it is common to supplement animal feed with low doses of antibiotics both for prophylaxis and growth promotion. Although the 40% MRSA contamination rate of pig carcasses observed in Hong Kong (Ho et al., 2012) is similar to reported rates in Europe (de Neeling et al., 2007), local isolates tend to be resistant to more non-beta-lactam antibiotics.

SGE technique detected a more resistant sub-population in a high percentage of strains. If these are indeed representative of populations with reduced vancomycin susceptibility, this is of concern. In clinical isolates, such heterogeneous strains have been associated with a rapid increase in vancomycin resistance in the presence of the drug and subsequent treatment failure (Rong and Leonard, 2010). Recognition of hVISA remains difficult and several studies have reported different outcomes for the various methods available for detection (Riederer et al., 2011; Satola et al., 2011). Whilst not being recognized as a reference method, SGE does allow easy visualization of sub-populations by 24 h in the form of trailing endpoints (Doddangoudar et al., 2010), which can be missed by other methods (Leonard et al., 2009; van Hal et al., 2011). Investigation of the five strains having an MIC > 2.0 mg/L and a TEC by SGE using phenotypic methods of hVISA recognition did not yield MIC values which met the definition of hVISA, but revealed presence of microcolonies in the clear areas for three by GRD and in all five by MET after a 48 h incubation period. Although the GRD/MET MIC results did not confirm the isolates as hVISA, the presence of microcolonies has been suggested to reflect the presence of more resistant individuals in the population (Howden et al., 2010). Further studies may help to determine if a TEC does correlate with presence of a more resistant sub-population.

The strains designated as vancomycin resistant by SGE were only marginally above the breakpoint and the evaluation of the method has shown a CV of 10% (Doddangoudar et al., 2010). However, this margin of error could move strains across the breakpoint as the vast majority were clustered around this level. This limitation of accuracy also applies to all MIC detection methods. It is of note that all three methods used showed clustering around the breakpoint indicating increases in resistance and results from AD closely matched those of SGE. Notably, BMD previously performed on these isolates (Ho et al., 2012) had not indicated any vancomycin resistance. However, several studies have reported that BMD may fail to detect vancomycin non-susceptible strains which are evident by Etest or AD (Walsh et al., 2001; Nadarajah et al., 2010; Vaudaux et al., 2010).

Porcine MRSA are widely distributed in the production chain and MRSA ST9 has been isolated locally from butchers (Boost et al., 2012) as well as from chopping boards (Lee et al., 2012), retail meats (Boost et al., 2013), and roasted pork (Young et al., 2012). Other studies on resistance of LA-MRSA have mainly focused on ST398, but none have reported resistance to vancomycin. The need for performing an MIC method to detect vancomycin resistance may discourage investigation of susceptibility and some studies still report use of disc diffusion methods for this agent. Alternatively, such resistance may be more typical of Asian isolates of ST9, which display more resistance to other non-beta-lactam antibiotics (Ho et al., 2012).

It has been demonstrated that a point mutation in *vra*S can lead to activation of *vra*R and development of hVISA (Cui et al., 2009) and that a mutation in *gra*R results in thickening of the cell wall and conversion of hVISA to VISA (Neoh et al., 2008). These changes have been observed in a local clinical isolate of VISA and in six MRSA strains which were induced with vancomycin (Doddangoudar et al., 2011, 2012). However, of the induced isolates with reduced susceptibility, changes in *vra*S were only present in two, one demonstrating a mutation in *vra*S, and a second having a stop codon. Similar changes have been noted in *vra*S in a clinical isolate of VISA (Doddangoudar et al., 2011). In the present study, seven of the 10 non-susceptible strains showed a mutation in *gra*R and two of these also had a stop codon as a result of a frame shift. One of the stop codons has previously been observed in induced strains (Doddangoudar et al., 2012). However, the lack of mutations in some of the non-susceptible porcine isolates suggests that changes in the VraSR/GraSR component systems alone may not explain the phenomenon of reduced susceptibility in these strains. Other workers have examined other regulatory genes in clinical isolates displaying reduced vancomycin susceptibility including *rpo*B, *walk*, and *clf*, but none of these has been shown to be present in all strains (Howden et al., 2011; Shoji et al., 2011; Watanabe et al., 2011; Hafer et al., 2012). It has been suggested that certain mechanisms may be more common in some clones or in some geographic regions (Hafer et al., 2012), with *vra*SR or *gra*SR having SNPs in ST8, whereas ST5 demonstrated changes in *wal*k.

However, an alternative explanation may be that the strains are in the process of losing vancomycin non-susceptibility. It has been demonstrated that strains which have been exposed to vancomycin become non-susceptible to this agent, but rapidly revert to susceptibility if the drug pressure is removed (Doddangoudar et al., 2012; Ludwig et al., 2012). This loss of susceptibility may be accompanied by appearance of STOP codons in the *vra*SR/*gra*SR two component systems (Doddangoudar et al., 2012). In this study, stop codons were present in *vra*S of one and in *gra*R of four porcine non-susceptible strains and interestingly in both genes of the strain used as a porcine negative control. It is therefore, possible that these strains were in the process of reversion to susceptibility in the absence of vancomycin and that the overall elevated MICs of the isolates reflects a population which is losing resistance.

This study has shown that LA-MRSA strains can display reduced susceptibility to vancomycin, even if currently MIC levels are only slightly above the breakpoint. Problems of treatment failure have been reported in clinical isolates with similar MICs and so caution with LA-MRSA is necessary. The lack of a consistent genetic marker for vancomycin low level resistance makes detection of these organisms difficult and there is a need for further investigation of changes in other regulatory systems in porcine isolates.

### Conflict of interest statement

The authors declare that the research was conducted in the absence of any commercial or financial relationships that could be construed as a potential conflict of interest.
